# Eye Gaze Based 3D Triangulation for Robotic Bionic Eyes

**DOI:** 10.3390/s20185271

**Published:** 2020-09-15

**Authors:** Di Fan, Yanyang Liu, Xiaopeng Chen, Fei Meng, Xilong Liu, Zakir Ullah, Wei Cheng, Yunhui Liu, Qiang Huang

**Affiliations:** 1 School of Mechatronical Engineering, Beijing Institute of Technology, Beijing 100081, China; fandi0126@bit.edu.cn (D.F.); bitdreamsky@bit.edu.cn (W.C.); 2 AIPARK, Zhangjiakou 075000, China; liuyanyang@aipark.com; 3 Beijing Advanced Innovation Center for Intelligent Robots and Systems, Beijing Institute of Technology, Beijing 100081, China; mfly0208@bit.edu.cn (F.M.); 7520190136@bit.edu.cn (Z.U.); qhuang@bit.edu.cn (Q.H.); 4 Key Laboratory of Biomimetic Robots and Systems (Beijing Institute of Technology), Ministry of Education, Beijing 100081, China; 5 Institute of Automation, Chinese Academy of Sciences, Beijing 100190, China; xilong.liu@ia.ac.cn; 6 Department of Mechanical and Automation Engineering, The Chinese University of Hong Kong, Shatin, NT, Hong Kong SAR 999077, China; yhliu@mae.cuhk.edu.hk

**Keywords:** active binocular vision, 3D coordinates estimation, eye gaze, 3D triangulation, robotic bionic eyes

## Abstract

Three-dimensional (3D) triangulation based on active binocular vision has increasing amounts of applications in computer vision and robotics. An active binocular vision system with non-fixed cameras needs to calibrate the stereo extrinsic parameters online to perform 3D triangulation. However, the accuracy of stereo extrinsic parameters and disparity have a significant impact on 3D triangulation precision. We propose a novel eye gaze based 3D triangulation method that does not use stereo extrinsic parameters directly in order to reduce the impact. Instead, we drive both cameras to gaze at a 3D spatial point ***P*** at the optical center through visual servoing. Subsequently, we can obtain the 3D coordinates of ***P*** through the intersection of the two optical axes of both cameras. We have performed experiments to compare with previous disparity based work, named the integrated two-pose calibration (ITPC) method, using our robotic bionic eyes. The experiments show that our method achieves comparable results with ITPC.

## 1. Introduction

Active binocular vision is a binocular vision system that could actively change its own view direction. It is beneficial for multiple applications such as manipulation [1], three-dimensional (3D) reconstruction [2], navigation [3], 3D mapping [4], and so on. 3D coordinates estimation based on active binocular vision attracts extensive research interests.

Active binocular vision systems can be divided into two categories: the first category has fixed cameras and the second category has non-fixed cameras. The vision systems of ASIMO [5], HRP-3 [6], HRP-4 [7], PR2 [8], ATLAS [9], and Walkman [10] belong to the first category. However, this category can not perceive objects that are very close to the cameras. The second category, which is similar as human vision system, can obviously improve flexibility and extend the field of view. The vision systems of the ESCHeR head [11], Yorick head [12], MAC-EYE robot eyes [13], iCub head [14], ARMAR-III head [15], Flobi head [16], Zhang XL’s robot eyes [17], ARMAR-4 head [18], Muecas [19], Romeo [20], SARA [21], and our designed anthropomorphic robotic bionic eyes [22,23,24] belong to the second category.

Usually, 3D triangulation can be performed using disparity and stereo extrinsic parameters [25,26,27,28,29,30,31]. The stereo extrinsic parameters are calibrated offline [32,33] or online [34,35,36,37,38,39]. Active vision systems of the first category only need offline calibration as the stereo extrinsic parameters are fixed, since one camera is static with respect to (w.r.t) the other. The stereo extrinsic parameters of the second category need to be calibrated online because the stereo extrinsic parameters may change all the time. In [40], Chen et al. proposed an integrated two-pose calibration (ITPC) method that calculated the stereo extrinsic parameters online using forward kinematics and the calibrated head-eye parameters.

**Figure 1:**
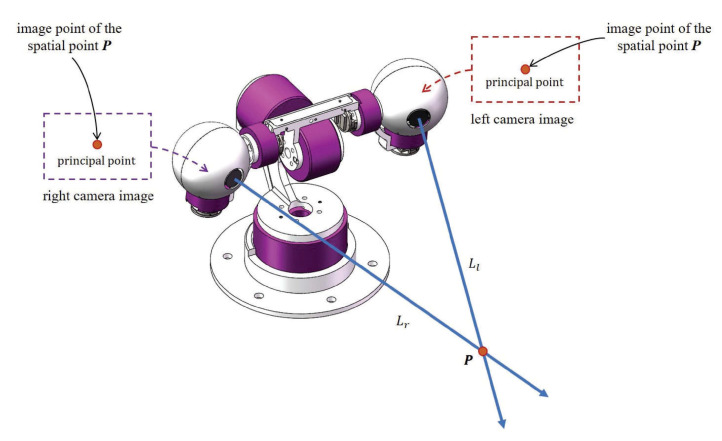
In our proposed method, we use eye gazing or visual servoing to actively adjust the view direction of each camera to keep the 2D image of a three-dimensional (3D) point P at the principal point. In other words, P is located at the fixation point. 3D triangulation could be performed through the intersection of two cameras’ optical axes.

However, in the disparity based triangulation method, the errors of the stereo extrinsic parameters and disparity have a significant impact on 3D triangulation [41]. In [41], Dang et al. analyzed that the stereo extrinsic parameters error can generate baseline error, pixel errors. The disparity error is generated by image detection and matching. The baseline error, pixel errors, and the disparity error will directly affect the 3D triangulation precision. In order to reduce the impact, this paper proposes a novel eye gaze based 3D triangulation method that does not use stereo extrinsic parameters directly. In our proposed method, the 2D image of a spatial point P in each camera is kept at the principal point through eye gazing or visual servoing. Or, the point P is always at the location of the fixation point of the two cameras. After that, we could obtain 3D coordinates of P through intersection of the two optical axes. In real applications, the fixation point P is represented by the middle point of the common perpendicular line segment of the two skew optical axes. Figure 1 shows the idea. The advantage of the eye gaze based triangulation is that we no longer need the stereo extrinsic parameters directly. The other advantage is that image detection error tolerance is higher than the disparity based triangulation method.

The main contribution of this paper is that keeping the target point lies on the optical axes of each camera through eye gazing, we can perform 3D triangulation through calculating the intersecting point of two optical axes of cameras instead of solving the projection equations of two cameras that rely on disparity and stereo extrinsic parameters.

## 2. System Configuration

**Figure 2:**
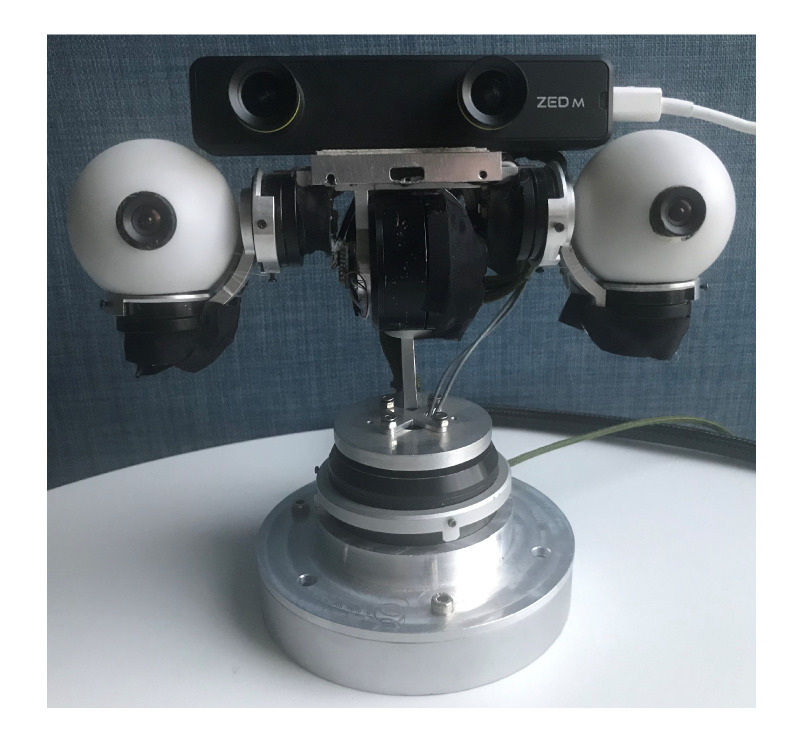
The anthropomorphic robotic bionic eyes.

**Figure 3:**
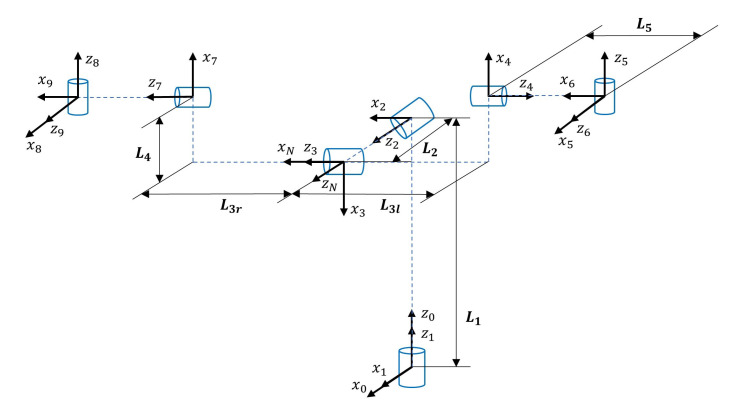
The definition of the robot bionic eyes’ coordinate system.

The anthropomorphic robotic bionic eyes we designed (as shown in Figure 2) for human-like active stereo perception, is illustrated in detail in [24]. This is a revised version with seven degree of freedoms (DOFs). It has two eyes and one neck. Each of the eye has two DOFs: tilt and pan. The neck has three DOFs. Joints of the neck and eyes are serially connected. It means that the links are connected serially by the neck and eyes joints and form ordered open chains. In the ordered open chain, the next joint is the load of the previous joint. As shown in Figure 3, our designed robotic bionic eyes contain two serial open chains: frame 0→1→2→3→4→5→6, frame 0→1→2→3→7→8→9. Each eye is installed with a USB camera with resolution of 640 × 480 @ 120 fps. A common trigger signal is connected to both cameras in order to realize hardware synchronization.

We define the coordinate systems that are based on the standard D-H convention. Figure 3 shows the frame definitions. Table 1 shows the D-H parameters of the robotic bionic eyes. The transformation matrix ${}^{i-1}T_{i}$ between Frame *i* and Frame i−1 can be calculated according to the D-H parameters [42].

## 3. Eye Gaze Based 3D Triangulation Method

The main idea of the eye gaze [43,44,45,46] based 3D triangulation method is to control the view direction of both cameras to keep a 3D spatial point P lies on the optical axes of both cameras. We are able to calculate the exact 3D coordinates of P through calculating the intersection point of the two optical axes, as we could obtain the pose of each camera through forward kinematics. We also know that once P lies on the optical axis of a camera, the image of P will be at the principal point of the 2D image. Accordingly, we could perform image based visual servoing to change the pose of both cameras to keep the images of P at the principal points of two cameras and, consequently, P lies on the two optical axes.

In this section, we first introduce visual servoing based approach for eye gazing, and then we show the process of calculation of the intersection point.

### 3.1. Visual Servoing Based Eye Gazing

We use image based visual servoing to control the pose of each camera. We will first get relationship of the velocity of the image feature points and the velocity of the joints. After that, we will present a simple visual servoing control law in order to generate commands to drive the feature point error down to zero.

#### 3.1.1. Relation Matrix

Here, we use a relation matrix to describe the velocity of the image feature points and the joint velocity. The relation matrix could be decomposed to be the multiplication of an Interaction matrix and a Jacobian matrix. An Interaction matrix is used to build the relationship of the velocity of an image feature point and the velocity of the camera. A Jacobian Matrix is used to describe the relationship of the velocity of the camera and joint velocity.

(1) $$\dot{p}=J_{image}V_{n},$$

where $\dot{p}=\;{\left[\dot{u}\;\dot{v}\right]}^{T}$ is the velocity of an image feature point, $V_{n}={\left[v_{x}\;v_{y}\;v_{z}\;\omega_{x}\;\omega_{y}\;\omega_{z}\right]}^{T}$ is the velocity of a camera, and $J_{image}$ is the Interaction matrix calculated, as follows:

(2) $$J_{image}=\left[\begin{array}{cccccc} -\frac{f_{u}}{Z_{c}} & 0 & \frac{u}{Z_{c}} & \frac{uv}{f_{u}} & -\frac{\left({f_{u}}^{2}+u^{2}\right)}{f_{u}} & v\\ 0 & -\frac{f_{v}}{Z_{c}} & \frac{v}{Z_{c}} & \frac{\left({f_{v}}^{2}+v^{2}\right)}{f_{v}} & -\frac{uv}{f_{v}} & -u \end{array}\right],$$

where $f_{u}$ and $f_{v}$ is the focal length in the *X* and *Y* directions of the camera, respectively, $Z_{c}$ is the depth of the spatial point P in the camera frame.

The relationship between $V_{n}$ and the joint velocity $\dot{\theta }$ could be described, as follows:

(3) $$V_{n}=J_{joint}\dot{\theta },$$

where $J_{joint}$ is the Jacobian matrix w.r.t camera frame, and it is defined as:

(4) $$J_{joint}=\left[\begin{array}{cc} R & 0\\ 0 & R \end{array}\right]J_{\theta },$$

where R is the rotation matrix of the base frame w.r.t the camera frame, and $J_{\theta }={\left[J_{\theta_{1}}\;J_{\theta_{2}}\;\ldots \;J_{\theta_{n}}\right]}^{T}$ is the Jacobian matrix w.r.t the base frame. The elements $J_{\theta_{i}}$ (i=1,2,…,n) can be calculated, as follows:

(5) $$J_{\theta_{i}}=\left[\begin{array}{c} Z_{i-1}\times \left(O_{n}-O_{i-1}\right)\\ Z_{i-1} \end{array}\right],$$

where $Z_{i}$ and $O_{i}$ are the direction vector of *Z* axis and the origin of Frame $O_{i}-x_{i}y_{i}z_{i}$ w.r.t base frame.

Accordingly, the relationship between the velocity of an image feature point $\dot{p}={\left[\dot{u}\;\dot{v}\right]}^{T}$ and the joint velocity $\dot{\theta }$ can be formulated, as follows:

(6) $$\dot{p}=J_{image}J_{joint}\dot{\theta }.$$

#### 3.1.2. Image Based Visual Servoing Control Law

We use a simple image based visual servoing control law to control the motion of the camera so as to reduce the error of the expected image point $p^{*}={\left[u^{*}\;v^{*}\right]}^{T}$ and the actual image point $p={\left[u\;v\right]}^{T}$ of 3D spatial point P, where ${\left[u^{*}\;v^{*}\right]}^{T}$ are set to the principal point ${\left[u_{0}\;v_{0}\right]}^{T}$, which can be achieved from the intrinsic parameters, and ${\left[u\;v\right]}^{T}$ are the actual pixel coordinates of the spatial point P in the camera.

(7) $$\dot{e}=-Ke,$$

where $K=diag\{k_{1},k_{2}\}$ is the gain matrix, and where $k_{1}$ and $k_{2}$ are the gain used to control how fast the pixel errors in the *X* and *Y* directions are regulated to zero independently [47], and $e=p^{*}-p={\left[u^{*}-u\;v^{*}-v\right]}^{T}$ is the error between $p^{*}$ and p. From Equation (7), we can infer that the error will decay to zero exponentially, where K is the exponential coefficient.

Because

(8) $$\dot{e}={\dot{p}}^{*}-\dot{p}=-\dot{p},$$

we could derive the following equation from Equations (6)–(8).

(9) $$J_{image}J_{joint}\dot{\theta }=K\left(p^{*}-p\right)=K\left[\begin{array}{c} u^{*}-u\\ v^{*}-v \end{array}\right].$$

The above equation could be rewritten for the left eye and right eye, respectively. For the left eye, we could derive

(10) $$J_{image_{l}}J_{joint_{l}}{\dot{\theta }}_{l}=J_{l}{\dot{\theta }}_{l}=K\left({p^{*}}_{l}-p_{l}\right)=K\left[\begin{array}{c} u_{l}^{*}-u_{l}\\ v_{l}^{*}-v_{l} \end{array}\right],$$

where $p_{l}={\left[u_{l}\;v_{l}\right]}^{T}$ and ${p^{*}}_{l}={\left[u_{l}^{*}\;v_{l}^{*}\right]}^{T}$ are the actual pixel coordinates and the expected pixel coordinates of the spatial point in the left camera, respectively, and ${\dot{\theta }}_{l}={\left[{\dot{\theta }}_{1}\;{\dot{\theta }}_{2}\;{\dot{\theta }}_{3}\;{\dot{\theta }}_{4}\;{\dot{\theta }}_{5}\right]}^{T}$ is the joint velocity.

We fix the neck joints and establish the base frame $O_{N}-x_{N}y_{N}z_{N}$ at link 3, the joint velocity vector is rewritten as:

(11) $${\dot{\theta }}_{l}={\left[\begin{array}{ccccc} 0 & 0 & 0 & {\dot{\theta }}_{4} & {\dot{\theta }}_{5} \end{array}\right]}^{T}.$$

From Equations (10) and (11), the velocities of left eye joints can be calculated, as follows:

(12) $$\left[\begin{array}{c} {\dot{\theta }}_{4}\\ {\dot{\theta }}_{5} \end{array}\right]={\left[\begin{array}{cc} J_{l_{14}} & J_{l_{15}}\\ J_{l_{24}} & J_{l_{25}} \end{array}\right]}^{-1}K\left({p^{*}}_{l}-p_{l}\right)=K\left[\begin{array}{c} u_{l}^{*}-u_{l}\\ v_{l}^{*}-v_{l} \end{array}\right],$$

where $J_{l_{ij}}$ is the element on the *i*-th row and *j*-th column in the matrix $J_{l}$.

Similarly, the velocities of the right eye joints can be calculated, as follows:

(13) $$\left[\begin{array}{c} {\dot{\theta }}_{7}\\ {\dot{\theta }}_{8} \end{array}\right]={\left[\begin{array}{cc} J_{r_{14}} & J_{r_{15}}\\ J_{r_{24}} & J_{r_{25}} \end{array}\right]}^{-1}K\left({p^{*}}_{r}-p_{r}\right)=K\left[\begin{array}{c} u_{r}^{*}-u_{r}\\ v_{r}^{*}-v_{r} \end{array}\right].$$

In each loop of the visual servoing, we send the generated eye joint velocity commands to drive the robotic bionic eyes to the view direction that reduces the error between $p^{*}$ and p in both cameras.

### 3.2. 3D Triangulation by Calculation of Intersection Point

After visual servoing, the point P will lie on the optical axes of each camera, and theoretically the intersection point of the two optical axes is the representation of P. We could calculate the intersection point geometrically if the equations of the two optical axes are obtained. We could use forward kinematics in order to derive the description of the two optical axes because each axis is the *Z* of the camera frame for each eye. In real situation, the two optical lines may not intersect each other, and so we use the middle point of the common perpendicular line segment of the two skew optical axes to be the representation of the intersection point instead.

#### 3.2.1. Calculation of the Coordinates of the Optical Axes

First of all, we will deduct the process to calculate the coordinates of the two optical axes $L_{l}$ and $L_{r}$. The optical axis of each camera is the coordinates of *Z* axis w.r.t the neck frame *N*, and so we need to derive the transformation matrix of the frame of the two cameras w.r.t frame *N* through forward kinematics, or the pose of left camera ${}^{N}T_{Cl}$ and the pose of right camera ${}^{N}T_{Cr}$.

(14) $${}^{N}T_{Cl}={}^{N}T_{6}{}^{6}T_{Cl},$$

where ${}^{6}T_{Cl}$ is the head-eye parameters of the left camera, which can be calculated using the method in [40]. ${}^{N}T_{6}$ is represented as the transformation matrix of the left eye frame w.r.t the neck frame *N*, and it can be obtained using the D-H parameters of the robotic bionic eyes, as follows:

(15) $${}^{N}T_{6}={}^{N}T_{3}{}^{3}T_{4}{}^{4}T_{5}{}^{5}T_{6}.$$

It is also the case for the right camera.

(16) $${}^{N}T_{Cr}={}^{N}T_{9}{}^{9}T_{Cr},$$

where ${}^{9}T_{Cr}$ is head-eye parameters of the right camera. ${}^{N}T_{9}$ is the transformation matrix of the right eye frame w.r.t the neck frame *N*, it can be obtained, as follows:

(17) $${}^{N}T_{9}={}^{N}T_{3}{}^{3}T_{7}{}^{7}T_{8}{}^{8}T_{9}.$$

Accordingly, we can perform real-time calculation of $L_{l}$ and $L_{r}$ using Equations (14) and (16).

#### 3.2.2. Calculation of the Intersection Point

**Figure 4:**
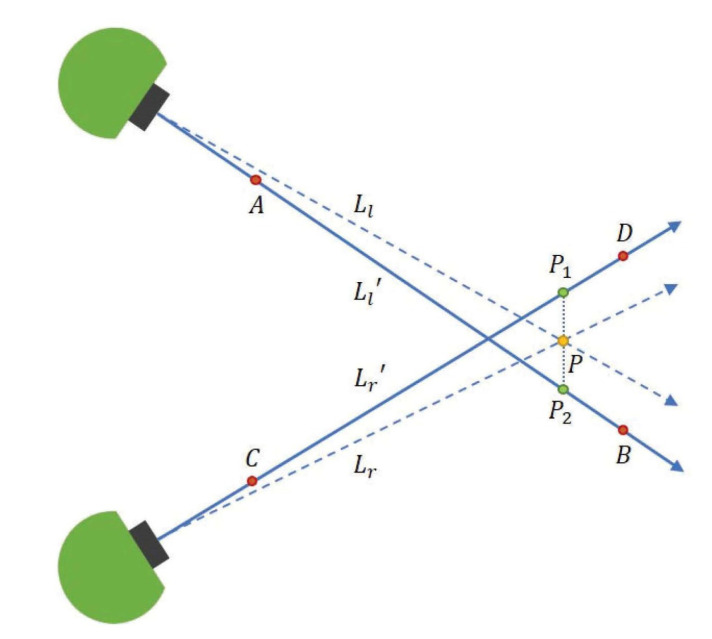
The actual model of our proposed method.

We are going to calculate the intersection point of the optical lines $L_{l}$ and $L_{r}$. Here, $L_{l}$ and $L_{r}$ are the ideal optical axes. The actual optical axes ${L_{l}}^{{}^{\prime }}$ and ${L_{r}}^{{}^{\prime }}$ will no longer intersect each other due to the measurement errors. It means that ${L_{l}}^{{}^{\prime }}$ and ${L_{r}}^{{}^{\prime }}$ may not in the same plane. We use the middle point of their common perpendicular line segment $P_{1}P_{2}$ to be the representation of P instead, which is shown in Figure 4.

In order to obtain P, we need to calculate $P_{1}$ and $P_{2}$. Here, $P_{1}$ is the intersection of line ${L_{r}}^{{}^{\prime }}$ and plane derived from ${L_{l}}^{{}^{\prime }}$ and $P_{1}P_{2}$. $P_{2}$ is the intersection of line ${L_{l}}^{{}^{\prime }}$ and plane derived from ${L_{r}}^{{}^{\prime }}$ and $P_{1}P_{2}$.

First of all, we would like to calculate the plane derived from ${L_{l}}^{{}^{\prime }}$ and $P_{1}P_{2}$. In order to do that, we will introduce two points C, D on line ${L_{r}}^{{}^{\prime }}$ with coordinates of ${}^{Cr}C={\left(0,0,10,1\right)}^{T}$ and ${}^{Cr}D={\left(0,0,20,1\right)}^{T}$ in the right camera frame. We set the coordinates in frame *N* as ${}^{N}C$ and ${}^{N}D$. And ${}^{N}C={}^{N}T_{Cr}{}^{Cr}C$ and ${}^{N}D={}^{N}T_{Cr}{}^{Cr}D$. We will also introduce two points A, B on line ${L_{l}}^{{}^{\prime }}$. The ${L_{r}}^{{}^{\prime }}$ in frame *N* (in the following statements, all of the parameters are represented in frame *N*) are defined, as follows:

(18) X=C+λ(D−C).

We use $n_{{L_{l}}^{{}^{\prime }}}$, $n_{{L_{r}}^{{}^{\prime }}}$, $n_{P_{1}P_{2}}$, $n_{P_{1}P_{2}{L_{l}}^{{}^{\prime }}}$ to represent the direction of ${L_{l}}^{{}^{\prime }}$, ${L_{r}}^{{}^{\prime }}$, $P_{1}P_{2}$ and the norm of plane $P_{1}P_{2}{L_{l}}^{{}^{\prime }}$ respectively. Accordingly,

(19) $$n_{P_{1}P_{2}}=n_{{L_{l}}^{{}^{\prime }}}\times n_{{L_{r}}^{{}^{\prime }}},$$

and

(20) $$n_{P_{1}P_{2}{L_{l}}^{{}^{\prime }}}=n_{{L_{l}}^{{}^{\prime }}}\times n_{P_{1}P_{2}},$$

and the plane $P_{1}P_{2}{L_{l}}^{{}^{\prime }}$ could be written as:

(21) $$n_{P_{1}P_{2}{L_{l}}^{{}^{\prime }}}\cdot \left(X-A\right)=0.$$

From Equations (18) and (21), we could get the intersection point $P_{1}$ between plane $P_{1}P_{2}{L_{l}}^{{}^{\prime }}$ and line ${L_{r}}^{{}^{\prime }}$.

(22) $$P_{1}=C+\frac{n_{P_{1}P_{2}{L_{l}}^{{}^{\prime }}}\cdot \left(A-C\right)}{n_{P_{1}P_{2}{L_{l}}^{{}^{\prime }}}\cdot \left(D-C\right)}\left(D-C\right).$$

Similarly, we could get $P_{2}$ like this:

(23) $$P_{2}=A+\frac{n_{P_{1}P_{2}{L_{r}}^{{}^{\prime }}}\cdot \left(C-A\right)}{n_{P_{1}P_{2}{L_{r}}^{{}^{\prime }}}\cdot \left(B-A\right)}\left(B-A\right).$$

Additionally, the fixation point *P* could be obtained:

(24) $$P=\frac{P_{1}+P_{2}}{2}.$$

Hence, the eye gaze based 3D triangulation Algorithm 1 can be summarized, as follows:

**Algorithm 1:** Eye Gaze Based 3D Triangulation Method.

**Visual Servoing based Eye Gazing:**
Gaze the 3D point P at the principal point in the left and right cameras simultaneously through visual servoing based eye gazing. In each loop of the visual servoing:Calculate the joint velocity commands of the robotic bionic eyes using Equations (12) and (13) according to pixel deviation in the left eye and right eye, respectively.Send the joint velocity commands to the robotic bionic eyes and get the feedback of joint angles.

**3D Triangulation by Calculation of Intersection Point:**
Calibrate the head-eye parameters ${}^{6}T_{Cl}$ and ${}^{9}T_{Cr}$ using method in [40].If visual servoing based eye gazing is successful:Calculate the transformation matrices ${}^{N}T_{6}$ and ${}^{N}T_{9}$ using the forward kinematics with joint position feedback.Obtain the optical axes of the left and right cameras using Equations (14) and (16).Calculate the 3D coordinates of P using Equations (22)–(24).



## 4. Propagation of Uncertainty

Uncertainty [48,49,50] is a quantification of the doubt about the validity of measurement results. The uncertainties of P using our method and the ITPC method are mainly propagated from image detection uncertainty. For our method, because some times the image uncertainty is tiny, which may result in no motion of optical axes because of the joint feedback precision ($\pm 0.04^{{}^{\circ}}$) is not as high as image coordinates. Subsequently, we could not measure the uncertainty directly, because there is no variation of the coordinates of P. In order to solve this problem, we use the law for the propagation of uncertainty [51,52] instead to calculate the theoretical uncertainties of point P obtained from the two methods that are based on image detection uncertainty.

### 4.1. Image Detection Uncertainty

The image detection uncertainty is represented as $u_{u}$ and $u_{v}$. They can be calculated, as follows:

(25) $$u_{u}=\sqrt{\frac{\sum_{i=1}^{n}\left(u_{i}-\bar{u}\right)}{n-1}},$$

(26) $$u_{v}=\sqrt{\frac{\sum_{i=1}^{n}\left(v_{i}-\bar{v}\right)}{n-1}},$$

where $u_{i}$, $v_{i}$ are the *i*-th (*i* = 1, 2, …, *n*) independent observation of the image coordinates *u*, *v*, respectively. $\bar{u}$, $\bar{v}$ are the average of the *n* observations of *u*, *v*, respectively.

### 4.2. Uncertainty of **P** Using Our Method

If eye gazing converges at time *t*, the image point of P coincide with the principal point in both cameras. After time *t*, the eye joint angles $\theta_{i}$ (*i* = 4, 5, 7, 8) will vary according to the variation of the image coordinates of P in both cameras through eye gazing. For the left eye, the joint angles $\theta_{4}$, $\theta_{5}$ can be formulated, as follows:

(27) $$\theta_{4}=arctan\left(\frac{v_{l}-v_{0_{l}}}{f_{v_{l}}}\right),$$

(28) $$\theta_{5}=arctan\left(\frac{u_{l}-u_{0_{l}}}{f_{u_{l}}}\right),$$

where $u_{l}$, $v_{l}$ are the image of P in the left camera, $u_{0_{l}}$, $v_{0_{l}}$ are the principal point in the left camera, and $f_{u_{l}}$, $f_{v_{l}}$ are the focal length in the *X* and *Y* directions of the left camera, respectively.

The uncertainties of the eye joint angles $\theta_{i}$ (*i* = 4, 5) can be calculated by propagating the uncertainties of the image of P in the left camera through partial derivatives of Equations (27) and (28), as follows:

(29) $$u_{\theta_{4}}=\frac{\partial \theta_{4}}{\partial v_{l}}u_{v_{l}},$$

(30) $$u_{\theta_{5}}=\frac{\partial \theta_{5}}{\partial u_{l}}u_{u_{l}},$$

where $u_{u_{l}}$, $u_{v_{l}}$ can be obtained using Equations (25) and (26). We use ${\bar{v}}_{l}$, ${\bar{u}}_{l}$ to be the replacement of $v_{l}$ and $u_{l}$ in $\frac{\partial \theta_{4}}{\partial v_{l}}$ and $\frac{\partial \theta_{5}}{\partial u_{l}}$. The uncertainties propagation of $\theta_{7}$, $\theta_{8}$ are similar to $\theta_{4}$, $\theta_{5}$.

From the previous Section, we derived that the 3D point P can be triangulated by Equations (22)–(24). The uncertainties of P can be calculated by propagating the uncertainties of the eye joint angles $\theta_{i}$ (*i* = 4, 5, 7, 8) through partial derivatives of Equation (24). The uncertainties of $P={\left(x_{p},y_{p},z_{p}\right)}^{T}$ using our proposed method can be formulated, as follows:

(31) $$u_{x_{p}}=\sqrt{{\left(\frac{\partial x_{p}}{\partial \theta_{4}}\right)}^{2}u_{\theta_{4}}^{2}+{\left(\frac{\partial x_{p}}{\partial \theta_{5}}\right)}^{2}u_{\theta_{5}}^{2}+{\left(\frac{\partial x_{p}}{\partial \theta_{7}}\right)}^{2}u_{\theta_{7}}^{2}+{\left(\frac{\partial x_{p}}{\partial \theta_{8}}\right)}^{2}u_{\theta_{8}}^{2}},$$

(32) $$u_{y_{p}}=\sqrt{{\left(\frac{\partial y_{p}}{\partial \theta_{4}}\right)}^{2}u_{\theta_{4}}^{2}+{\left(\frac{\partial y_{p}}{\partial \theta_{5}}\right)}^{2}u_{\theta_{5}}^{2}+{\left(\frac{\partial y_{p}}{\partial \theta_{7}}\right)}^{2}u_{\theta_{7}}^{2}+{\left(\frac{\partial y_{p}}{\partial \theta_{8}}\right)}^{2}u_{\theta_{8}}^{2}},$$

(33) $$u_{z_{p}}=\sqrt{{\left(\frac{\partial z_{p}}{\partial \theta_{4}}\right)}^{2}u_{\theta_{4}}^{2}+{\left(\frac{\partial x_{p}}{\partial \theta_{5}}\right)}^{2}u_{\theta_{5}}^{2}+{\left(\frac{\partial z_{p}}{\partial \theta_{7}}\right)}^{2}u_{\theta_{7}}^{2}+{\left(\frac{\partial z_{p}}{\partial \theta_{8}}\right)}^{2}u_{\theta_{8}}^{2}}.$$

In the above equations, we use ${\bar{\theta }}_{fb_{i}}$ or the average of the *n* independent repeated observations of the *i*-th (*i* = 4, 5, 7, 8) joint angle feedback as the value of $\theta_{i}$ in $\frac{\partial x_{p}}{\partial \theta_{i}}$, $\frac{\partial y_{p}}{\partial \theta_{i}}$, and $\frac{\partial z_{p}}{\partial \theta_{i}}$.

### 4.3. Uncertainty of **P** Using ITPC Method

In the ITPC method, the eye joint angles $\theta_{i}$ (*i* = 4, 5, 7, 8) will not change after time *t*. The stereo extrinsic parameters ${}^{Cr}T_{Cl}$ can be calculated using the eye joint angles $\theta_{i}$ (*i* = 4, 5, 7, 8). Here, $\theta_{i}=\theta_{fb_{i}}$, where $\theta_{fb_{i}}$ is the *i*-th joint angle feedback at time *t*. $P=(x_{p},y_{p},z_{p})$ can be calculated while using the intrinsic parameters of both cameras, the stereo extrinsic parameters ${}^{Cr}T_{Cl}$, and the image coordinates of P in both cameras. The uncertainties of P using the ITPC method can be calculated by propagating the uncertainties of the image coordinates in both cameras through partial derivatives:

(34) $$u_{x_{p}}=\sqrt{{\left(\frac{\partial x_{p}}{\partial u_{l}}\right)}^{2}u_{u_{l}}^{2}+{\left(\frac{\partial x_{p}}{\partial v_{l}}\right)}^{2}u_{v_{l}}^{2}+{\left(\frac{\partial x_{p}}{\partial u_{r}}\right)}^{2}u_{u_{r}}^{2}+{\left(\frac{\partial x_{p}}{\partial v_{r}}\right)}^{2}u_{v_{r}}^{2}},$$

(35) $$u_{y_{p}}=\sqrt{{\left(\frac{\partial y_{p}}{\partial u_{l}}\right)}^{2}u_{u_{l}}^{2}+{\left(\frac{\partial y_{p}}{\partial v_{l}}\right)}^{2}u_{v_{l}}^{2}+{\left(\frac{\partial y_{p}}{\partial u_{r}}\right)}^{2}u_{u_{r}}^{2}+{\left(\frac{\partial y_{p}}{\partial v_{r}}\right)}^{2}u_{v_{r}}^{2}},$$

(36) $$u_{z_{p}}=\sqrt{{\left(\frac{\partial z_{p}}{\partial u_{l}}\right)}^{2}u_{u_{l}}^{2}+{\left(\frac{\partial z_{p}}{\partial v_{l}}\right)}^{2}u_{v_{l}}^{2}+{\left(\frac{\partial z_{p}}{\partial u_{r}}\right)}^{2}u_{u_{r}}^{2}+{\left(\frac{\partial z_{p}}{\partial v_{r}}\right)}^{2}u_{v_{r}}^{2}},$$

where $u_{u_{l}}$, $u_{u_{r}}$ can be calculated using Equation (25). $u_{v_{l}}$, $u_{v_{r}}$ can be calculated using Equation (26). In $\frac{\partial x_{p}}{\partial u_{l}}$, $\frac{\partial x_{p}}{\partial v_{l}}$, $\frac{\partial x_{p}}{\partial u_{r}}$, $\frac{\partial x_{p}}{\partial v_{r}}$, $\frac{\partial y_{p}}{\partial u_{l}}$, $\frac{\partial y_{p}}{\partial v_{l}}$, $\frac{\partial y_{p}}{\partial u_{r}}$, $\frac{\partial y_{p}}{\partial v_{r}}$, $\frac{\partial z_{p}}{\partial u_{l}}$, $\frac{\partial z_{p}}{\partial v_{l}}$, $\frac{\partial z_{p}}{\partial u_{r}}$, and $\frac{\partial z_{p}}{\partial v_{r}}$, we use $u_{l}={\bar{u}}_{l}$, $v_{l}={\bar{v}}_{l}$, $u_{r}={\bar{u}}_{r}$, $v_{r}={\bar{v}}_{r}$.

## 5. Experiments and Results

We will first compare the triangulation performance of our method with ITPC method in simulated and physical experiments in order to evaluate the eye gaze based 3D triangulation method. After that, we will do the precision experiments comparing with stereo system ZED mini with fixed cameras. Finally, we will execute experiments to obtain the time response of our method.

### 5.1. Comparison Experiments with the ITPC Method

We will compare our proposed method with the ITPC method in both simulated and physical experiments. Static target points as well as moving target points will be triangulated to see the performance of the two methods.

#### 5.1.1. Simulated Experiments

We will do the two comparison experiment in simulation environments. The first is triangulation of static target points and the other is triangulation of a moving target point.

##### Experimental Setup

In simulated experiments, we use GAZEBO [53] in order to simulate the kinematics of the robotic bionic eyes with the same mechanical model as the physical robotic bionic eyes. We also use the same joint controller under the ROS framework to send joint velocity commands to GAZEBO and get the joint angle feedback as well as image feedback from both eyes.

**Figure 5:**
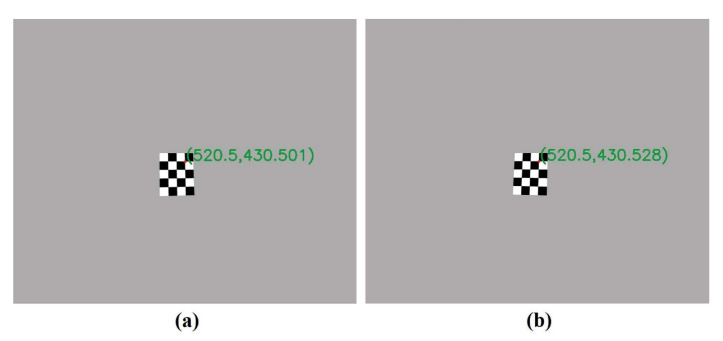
The images of P in the left and right simulated cameras. (**a**) left image; (**b**) right image.

The image size of the left and right cameras is 1040 × 860 in pixel. The intrinsic parameters obtained while using Bouguet toolbox [54] are shown in Table 2. The checkerboard corner points are detected with sub-pixel position accuracy. We choose the point at the upper right corner as the spatial point P. The expected pixel coordinates of point P in both cameras are fixed at the principal point (520.50, 430.50). Figure 5 shows the images of P in the left and right cameras.

##### Triangulation of Static Target Points

In this experiment, we get 21 different static target points by placing the simulated checkerboard at 21 different positions. At each position, we recorded the estimated 3D coordinates w.r.t the base frame *N* using our proposed method and ITPC method, respectively. The ground truth can be obtained from GAZEBO (as shown in Table 3).

**Figure 6:**
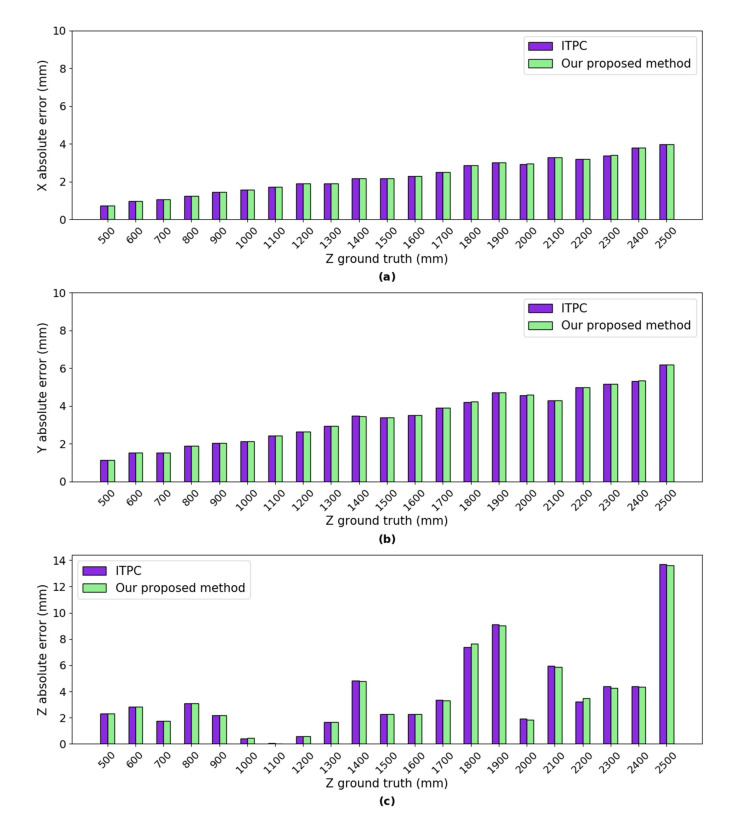
The absolute error of the mean of the estimated 3D coordinates using our proposed method and integrated two-pose calibration (ITPC) method w.r.t ground truth respectively. (**a**) absolute error in *X* axis; (**b**) absolute error in *Y* axis; and,(**c**) absolute error in *Z* axis.

***Error Analysis:*** we calculated the absolute errors of the mean of the 3D coordinates using our proposed method and ITPC method (as shown in Figure 6) w.r.t the ground truth, respectively. The absolute error of both methods in the simulated experiments are mainly from the error of D-H parameters, the error of intrinsic parameters and the error of head-eye parameters. The absolute errors in *X* axis of both methods are between 0.73 mm and 3.96 mm, as shown in Figure 6. The absolute errors in *Y* axis of both methods are between 1.14 mm and 6.18 mm. We can see that the minimum absolute errors in *Z* axis of both methods are only 0.02 mm and 0.04 mm, respectively, when *Z* ground truth is 1100 mm. The maximum absolute errors in *Z* axis of both methods only reach 0.5% of the ground truth depth. In conclusion, the absolute errors of our proposed method are very close to the ITPC method in the *X*, *Y*, and *Z* axes.

**Figure 7:**
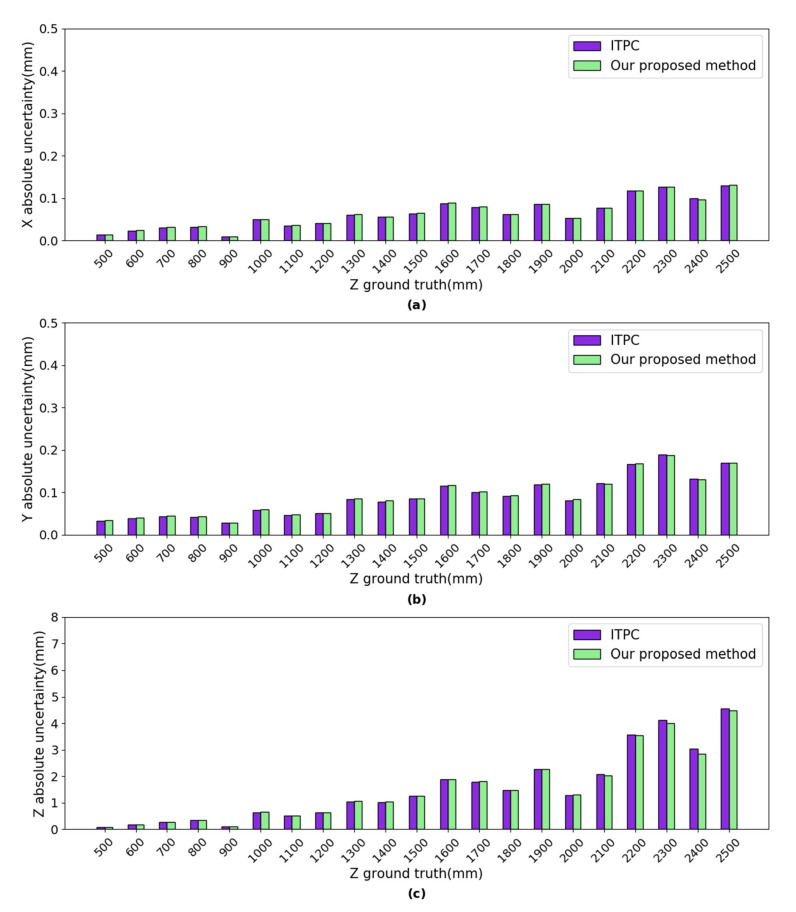
The uncertainties of our proposed method and ITPC method. (**a**) absolute uncertainty in *X* axis; (**b**) absolute uncertainty in *Y* axis; and, (**c**) absolute uncertainty in *Z* axis.

***Uncertainty Analysis:*** the uncertainties of our proposed method (shown in Figure 7) were calculated using Equations (31)–(33). The uncertainties of the ITPC method (shown in Figure 7) were calculated using Equations (34)–(36). At each position, the times *n* of independent repeated observations is 1150. For both of the left and right cameras, the image detection uncertainties in the *X* direction are between 0.006 pixels and 0.057 pixels. The image detection uncertainties in the *Y* direction are between 0.006 pixels and 0.057 pixels. The absolute uncertainties of our proposed method are very close to the ITPC method in the *X*, *Y* and *Z* axes, as shown in Figure 7. The absolute uncertainty in *X* axis of both methods are between 0.009 mm and 0.130 mm. The absolute uncertainty in *Y* axis of both methods are between 0.028 mm and 0.188 mm. The absolute uncertainty in *Z* axis of both methods are between 0.088 mm and 4.545 mm.

##### Triangulation of a Moving Target Point

We want to verify the effectiveness of our proposed method in the case that the target point is moving.

We placed a checkerboard at 1000 mm on the *Z* axis w.r.t the base frame *N*. The trajectory of the target point on the *X* and *Y* axes w.r.t frame *N* is x=r∗sin(t/10000) and y=−100+r∗cos(t/10000), respectively, where r=100 mm. We recorded the 3D coordinates of the moving target points estimated by our proposed method and ITPC method, respectively.

**Figure 8:**
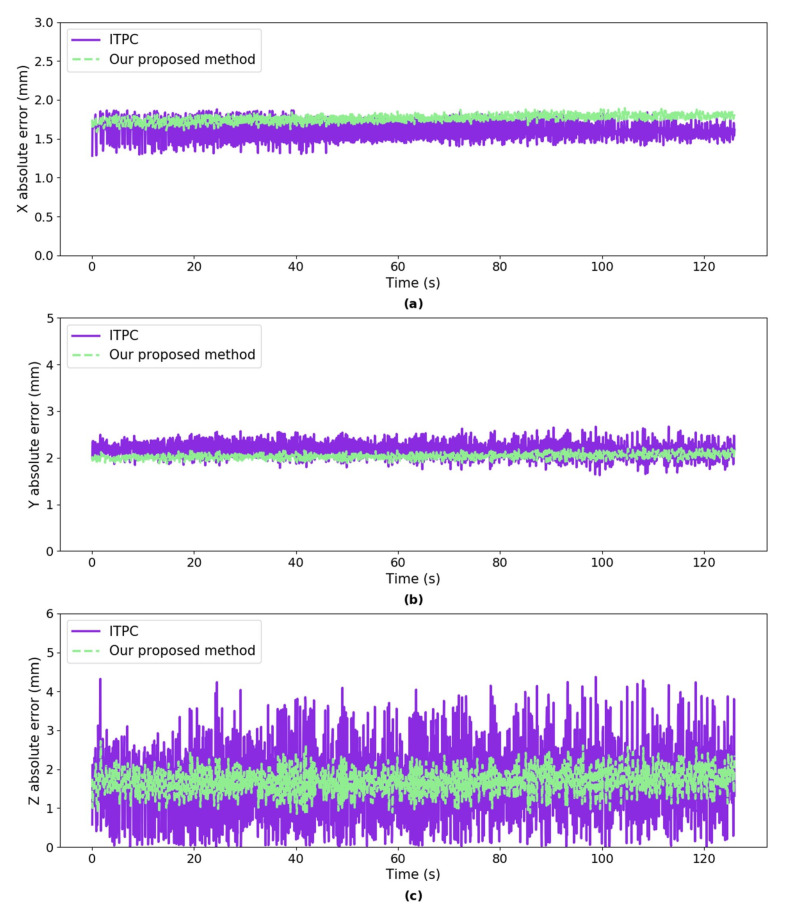
The absolute error of the mean of the estimated 3D coordinates using our proposed method and ITPC method w.r.t ground truth, respectively. (**a**) absolute error in *X* axis; (**b**) absolute error in *Y* axis; and, (**c**) absolute error in *Z* axis.

***Error Analysis:*** we calculated the absolute error of our proposed method and ITPC method w.r.t ground truth, respectively. The mean absolute error in *X* axis of both methods are 1.76 mm and 1.61 mm, respectively, as shown in Figure 8. The mean absolute error in *Y* axis of both methods are 2.03 mm and 2.17 mm, respectively. The mean absolute error in *Z* axis of both methods are 1.69 mm and 1.71 mm which reach only 0.17% of the ground truth depth. The mean absolute error in *X* axis of our proposed method is larger than the ITPC method and the mean absolute error in *Y*, *Z* axes of the proposed method are smaller than the ITPC method.

***Uncertainty Analysis:*** we calculated the standard deviation of the coordinates in *Z* axis as the uncertainties of our proposed method and ITPC method. In the experiments, the number *n* is 3780. The absolute uncertainties of our proposed method and the ITPC method in *Z* axis are 0.30 mm and 0.98 mm, respectively. The absolute uncertainties in *Z* axis of our proposed method are smaller than the ITPC method.

#### 5.1.2. Physical Experiments

The physical experiments are performed on the real robotic bionic eyes. Triangulation on static and moving target points is performed. Comparisons are carried out between our proposed method and the ITPC method.

##### Experimental Setup

**Figure 9:**
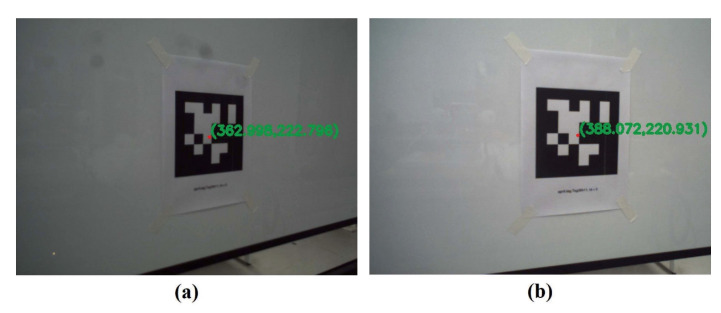
The images of the left and right cameras. (**a**) left image; (**b**) right image.

In physical experiments, the image size of the left and right cameras is 640 × 480 in pixel. The intrinsic parameters of both cameras are shown in Table 4. The distortion coefficients of the left and right cameras are [−0.0437, 0.1425, 0.0005, −0.0012, 0.0000] and [−0.0425, 0.1080, 0.0001, −0.0015, 0.0000], respectively. The maximum horizontal and vertical field of view of the real robotic bionic eyes are 170° and 170°, respectively. The AprilTag [55] information is obtained through ViSP [56] library. We choose the center (point P) of the AprilTag as the fixation point. The expected image of point P in the left and right cameras are set to the principal points (362.94, 222.53) and (388.09, 220.82), respectively. Figure 9 shows the images of P in the left and right cameras.

##### Triangulation of Static Target Points

We placed the AprilTag at 21 different positions (as shown in Table 5). At each position, the estimated 3D coordinates using our proposed method and ITPC method were recorded, respectively.

**Figure 10:**
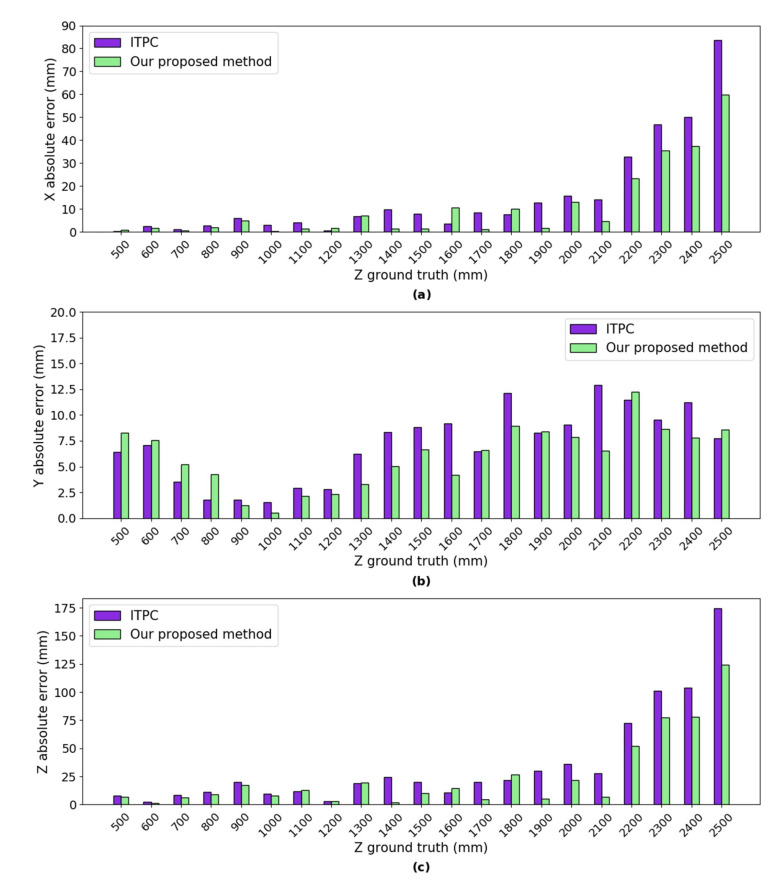
The absolute error of the mean of the estimated 3D coordinates using our proposed method and ITPC method w.r.t ground truth respectively. (**a**) absolute error in *X* axis; (**b**) absolute error in *Y* axis; and, (**c**) absolute error in *Z* axis.

***Error Analysis:*** we calculated the absolute error of the mean of estimated 3D coordinates w.r.t ground truth using our proposed method and ITPC method, respectively (as shown in Figure 10). The absolute error of both methods in the physical experiments are mainly from errors of forward kinematics, errors of intrinsic parameter, errors of head-eye parameters and joint offset errors. As shown in Figure 10, our proposed method are closer to the ground truth than the ITPC methods in the *X* and *Z* axes in most of the 21 different positions, especially when *Z* ground truth are larger than 2000 mm. The absolute error in *Y* axis of our proposed method is between 0.55 mm and 12.28 mm. The absolute error in *Y* axis of the ITPC method are between 1.52 mm and 12.88 mm. The minimum absolute errors in *Z* axis of our proposed method and the ITPC method are 1.42 mm and 2.32 mm, which reach 0.23% and 0.38% of the ground truth depth, respectively. The maximum absolute error in *Z* axis of our proposed method and the ITPC method are 124.49 mm and 174.64 mm, which reach 4.97% and 6.98% of the ground truth depth, respectively. Our proposed method obtains smaller mean absolute errors in the *X*, *Y*, and *Z* axes.

**Figure 11:**
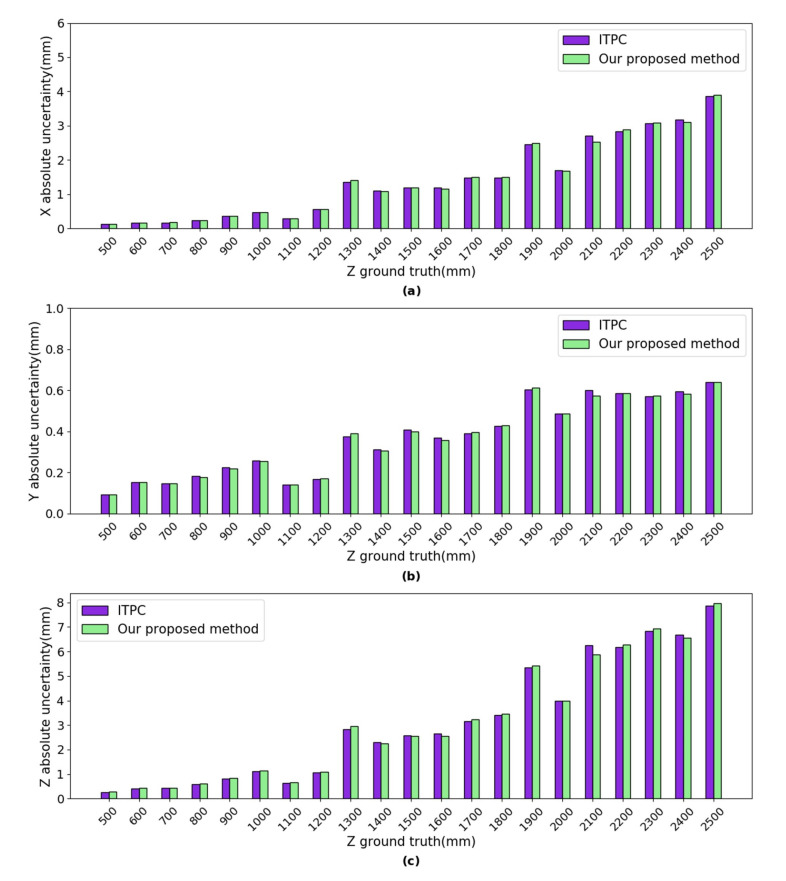
The uncertainties of our proposed method and ITPC method. (**a**) absolute uncertainty in *X* axis; (**b**) absolute uncertainty in *Y* axis; and, (**c**) absolute uncertainty in *Z* axis.

***Uncertainty Analysis:*** the uncertainties of our proposed method (shown in Figure 11) were calculated using Equations (31)–(33). The uncertainties of the ITPC method (shown in Figure 11) were calculated using Equations (34)–(36). At each position, *n* = 1200. For the left camera, the image detection uncertainties in the *X* direction are between 0.027 pixels and 0.089 pixels. The image detection uncertainties in the *Y* direction are between 0.039 pixels and 0.138 pixels. For the right camera, the image detection uncertainties in the *X* direction are between 0.016 pixels and 0.067 pixels. The image detection uncertainties in the *Y* direction are between 0.046 pixels and 0.138 pixels. The absolute uncertainty in *X* axis of both methods are between 0.119 mm and 3.905 mm, as shown in Figure 11. The absolute uncertainty in *Y* axis of both methods are between 0.091 mm and 0.640 mm. The absolute uncertainty in *Z* axis of both methods are between 0.268 mm and 7.975 mm. In conclusion, the absolute uncertainties of our proposed method are very close to the ITPC method in the *X*, *Y*, and *Z* axes.

##### Triangulation of a Moving Target Point

**Figure 12:**
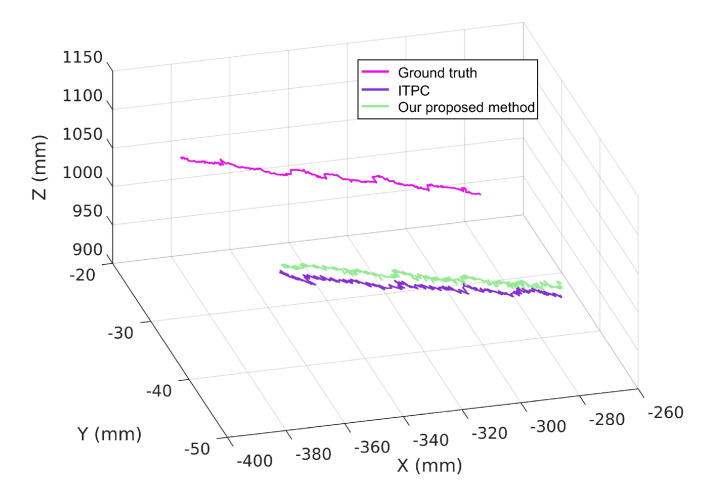
The trajectory of the ground truth and the estimated point P using our proposed method and ITPC method.

We move the target point of the AprilTag from (−278.60, −23.13, 957.84) to (−390.02, −30.39, 1111.91) at the average speed of 0.01 m/s. Figure 12 shows the trajectory of the ground truth and the estimated P using our proposed method and ITPC method.

**Figure 13:**
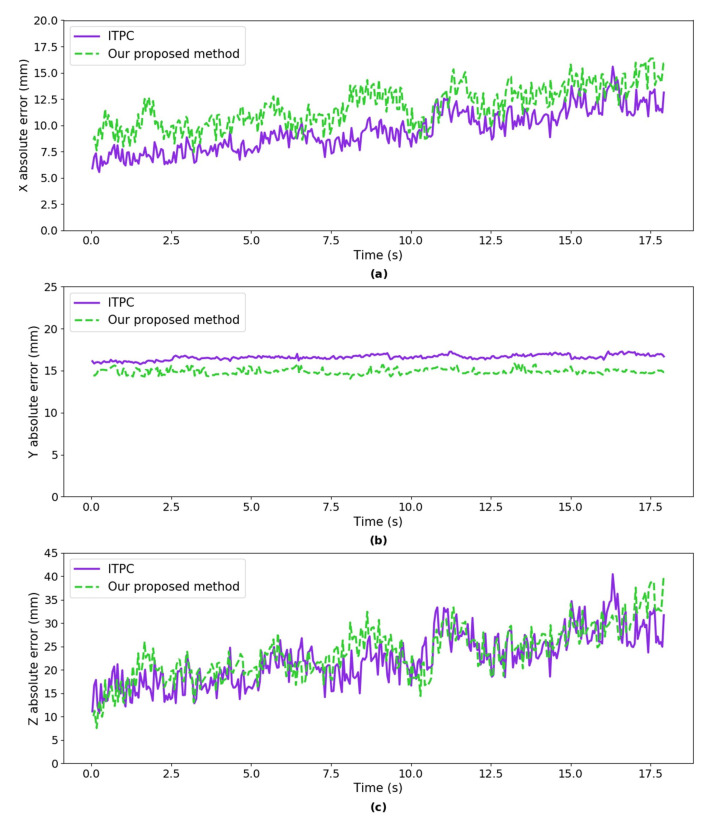
The absolute error of our proposed method and ITPC method w.r.t ground truth respectively. (**a**) absolute error in *X* axis; (**b**) absolute error in *Y* axis; and, (**c**) absolute error in *Z* axis.

***Error Analysis:*** we calculated the absolute error of our proposed method and ITPC method w.r.t ground truth respectively (shown in Figure 13). The mean absolute error in *X* axis of our proposed method and the ITPC method are 11.81 mm and 9.53 mm, as shown in Figure 13. The mean absolute error in *Y* axis of our proposed method and the ITPC method are 14.89 mm and 16.59 mm. The mean absolute error in *Z* axis of our proposed method and the ITPC method are 23.74 mm and 22.48 mm. The mean absolute error in *X*, *Z* axes of the proposed method are larger than the ITPC method, and the mean absolute error in *Y* axis of our proposed method is smaller than the ITPC method.

### 5.2. Comparison Experiments with Zed Mini

We compare the triangulation performance of our proposed method and ITPC method with stereo system ZED Mini while using fixed cameras (shown in Figure 2).

#### Experimental Setup

The image size of the left and right cameras of ZED mini are 1280 × 720 pixels. The intrinsic parameters including the focal length and principal point of both cameras are shown in Table 6. The maximum horizontal and vertical field of view of ZED mini are 90° and 60°, respectively. We placed the AprilTag at eight different positions to get 8 different spatial points. The estimated 3D coordinates using ZED mini are shown in Table 7.

**Figure 14:**
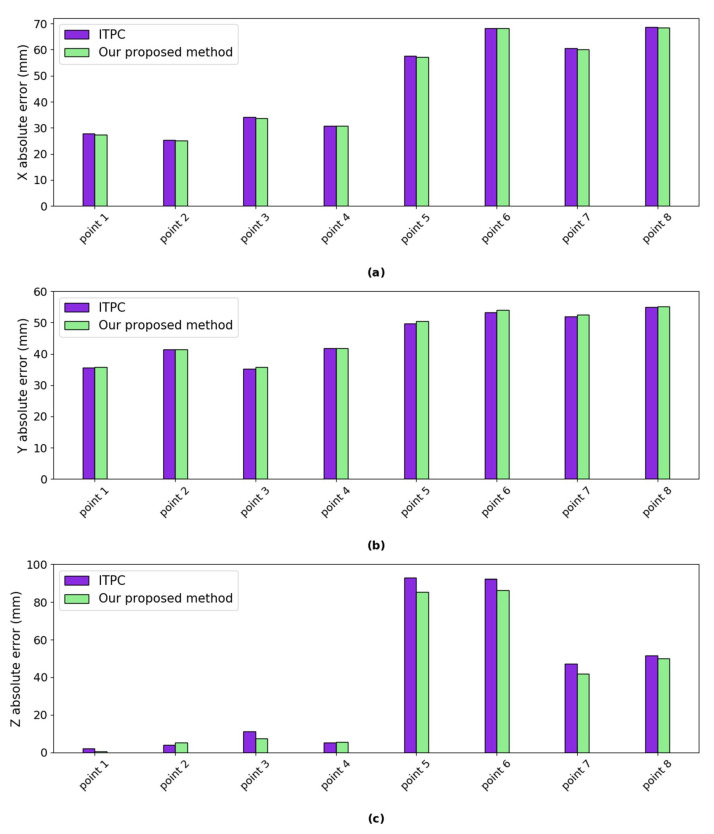
The absolute error of our proposed method and ITPC method w.r.t ZED Mini. (**a**) absolute error in *X* axis; (**b**) absolute error in *Y* axis; and, (**c**) absolute error in *Z* axis.

***Error analysis:*** we calculated the absolute error of our proposed method and ITPC method w.r.t ZED mini, respectively. As shown in Figure 14, the absolute errors of our proposed method are very close to the ITPC method in the *X* and *Y* axes. Our proposed method gets smaller absolute error in the *Z* axis at most of the eight different positions. The absolute error in *X* axis of both methods are between 25.17 mm and 68.63 mm. The absolute error in *Y* axis of both methods are between 35.26 mm and 55.18 mm. The minimum absolute errors in *Z* axis of our proposed method and ITPC method are 0.44 mm and 2.10 mm. The maximum absolute errors in *Z* axis of our proposed method and ITPC method are 86.40 mm and 92.90 mm. No camera movements exist in ZED mini. Our proposed method and ITPC method get a minimally larger errors when comparing with ZED mini mainly because of eye motion.

### 5.3. Time Performance Experiments

#### 5.3.1. Simulated Experiments

**Figure 15:**
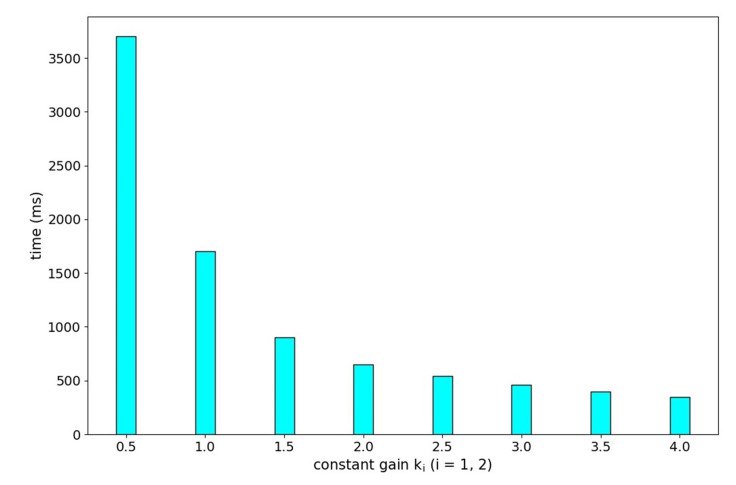
The time it takes for eye gazing affected by $k_{1}$ and $k_{2}$.

We perform experiments to evaluate how $k_{1}$ and $k_{2}$ in the constant coefficient matrix K affect the time it takes for eye gazing. We initially placed the simulated checkerboard to set the target point at (−0.00, −100.00, 1000). A step signal with amplitude of 100 mm in *Y* direction was applied to the target point. We adjusted $k_{1}$ and $k_{2}$ from 0.5 to 4.0. The system overshot when $k_{1}$ or $k_{2}$ is larger than 4.0. Increasing $k_{1}$ and $k_{2}$ moderately can shorten the time it takes for eye gazing, as shown in Figure 15.

#### 5.3.2. Physical Experiments

In the physical experiments, we placed the AprilTag to set the target point at (−0.00, −145.00, 1500). We set the robotic bionic eyes to the initial state. We set $k_{1}=4.0$, $k_{2}=4.0$ in K. It takes 650 ms to move the target point P from (385.98, 196.60) to (362.94, 222.53) in the left image frame, and from (361.09, 191.82) to (388.09, 220.82) in the right image frame, respectively, through eye gazing.

## 6. Conclusions

In this paper, we have proposed an eye gaze based 3D triangulation method for our designed robotic bionic eyes. The eye gaze was realized through image based visual servoing in order to keep the two optical axes pass through the target point P. We could obtain the 3D coordinates of P through the intersection of the two optical axes of both cameras. The optical axes of both cameras could be derived from forward kinematics with head-eye parameters calibrated beforehand. In real applications, the two optical axes may not intersect each other due to visual servoing errors and model errors, and so we use the middle point of the common perpendicular line segment of the two skew optical axes as the representation of the intersection point P.

From the simulated and physical experiments, we can see that the proposed method achieves comparable results with the ITPC method in the absolute errors and the propagated uncertainties, and our proposed method gets smaller mean absolute errors with the triangulation of static target points in physical experiments. Our proposed method and ITPC method get larger errors w.r.t conventional stereo systems with fixed cameras such as ZED Mini due to model errors introduced by manufacturing which include link length error, coaxiality error and error due to link stiffness. The experiments show that at the beginning of the visual servoing process our method need several hundred milliseconds to locate a target point to its optical center. Selecting $k_{1}$ and $k_{2}$ in the gain matrix K by using fuzzy PID could be potential solution to minimize the initial processing time to lacate a target point to its optical center.

Although our method has only tiny improvement in triangulation precision compare with the ITPC method, it is a new bionic approach for triangulation using eye gazing through image based visual servoing. Our system has much larger field of view than traditional stereo pair, such as ZED Mini, and it does not rely on stereo extrinsic parameters directly. Another advantage is that image detection error tolerance is higher. In the future, we are going to reduce the model error and joint offset error introduced by manufacturing to obtain compatible precision as stereo pair with fixed cameras.

## Figures and Tables

**Table 1:** The D-H parameters of the robotic bionic eyes. (Here parameters $d_{i}$, $\theta_{i}$, $a_{i}$, $\alpha_{i}$ are the link offset, joint angle, link length and link twist, respectively. The *i*-th joint offset is the value of $\theta_{i}$ in the initial state. $L_{1}$ is 64.27 mm, $L_{2}$ is 11.00 mm, $L_{3l}$ is 44.80 mm, $L_{3r}$ is 47.20 mm, $L_{4}$ is 13.80 mm, and $L_{5}$ is 30.33 mm).

*i*	$d_{i}/$mm	$\theta_{i}/$rad	$a_{i}/$mm	$\alpha_{i}/$rad	*i*-th joint offset/rad
1	0	$\theta_{1}$	0	0	0
2	$L_{1}$	$\theta_{2}$	0	−pi/2	−pi/2
3	$L_{2}$	$\theta_{3}$	0	pi/2	pi/2
4	$-L_{3l}$	$\theta_{4}$	$L_{4}$	pi	pi
5	$L_{5}$	$\theta_{5}$	0	pi/2	pi/2
6	0	0	0	−pi/2	−pi/2
7	$L_{3r}$	$\theta_{7}$	$L_{4}$	0	pi
8	$L_{5}$	$\theta_{8}$	0	−pi/2	−pi/2
9	0	0	0	−pi/2	−pi/2

**Table 2:** The intrinsic parameters of the left and right simulated cameras.

Camera	$f_{u}/$Pixel	$f_{v}/$Pixel	$u_{0}/$Pixel	$v_{0}/$Pixel
Left	619.71	619.71	520.50	430.50
Right	619.71	619.71	520.50	430.50

**Table 3:** The ground truth of the 3D coordinates of the 21 static target points.

Number	x/mm	y/mm	z/mm	Number	x/mm	y/mm	z/mm
1	−0.00	−99.81	499.98	12	−0.01	−99.84	1599.99
2	−0.00	−99.81	599.99	13	−0.01	−99.85	1699.99
3	−0.00	−99.81	699.99	14	−0.01	−99.85	1799.99
4	−0.00	−99.82	799.99	15	−0.01	−99.84	1899.99
5	−0.00	−99.82	899.99	16	−0.01	−99.86	1999.99
6	−0.01	−99.83	999.99	17	−0.01	−99.86	2099.99
7	−0.00	−99.83	1099.99	18	−0.01	−99.85	2199.99
8	−0.01	−99.83	1199.99	19	−0.01	−99.86	2299.99
9	−0.00	−99.84	1299.99	20	−0.01	−99.86	2399.99
10	−0.01	−99.84	1399.99	21	−0.01	−99.87	2499.99
11	−0.01	−99.85	1499.99				

**Table 4:** The intrinsic parameters of the left and right cameras of the designed robotic bionic eyes.

Camera	$f_{u}/$Pixel	$f_{v}/$Pixel	$u_{0}/$Pixel	$v_{0}/$Pixel
Left	521.66	521.83	362.94	222.53
Right	521.20	521.51	388.09	220.82

**Table 5:** The ground truth of the static target points.

Number	x/mm	y/mm	z/mm	Number	x/mm	y/mm	z/mm
1	−208.73	−177.64	508.04	12	−721.54	−180.49	1603.42
2	−232.55	−177.53	605.69	13	−754.63	−182.21	1704.31
3	−255.58	−176.02	699.53	14	−779.88	−181.77	1798.82
4	−324.48	−179.81	800.92	15	−816.46	−182.52	1909.67
5	−407.39	−178.42	899.89	16	−842.48	−182.31	2004.67
6	−452.88	−179.90	1001.11	17	−909.75	−171.71	2097.57
7	−486.87	−180.58	1102.26	18	−984.94	−168.96	2200.87
8	−563.40	−179.15	1198.49	19	−1029.85	−171.79	2296.25
9	−607.89	−181.18	1308.29	20	−1144.02	−168.11	2407.59
10	−649.50	−179.74	1404.37	21	−1229.31	−166.47	2506.59
11	−688.94	−182.54	1501.91				

**Table 6:** The intrinsic parameters of the left and right cameras of ZED mini.

Camera	$f_{u}/$Pixel	$f_{v}/$Pixel	$u_{0}/$Pixel	$v_{0}/$Pixel
Left	676.21	676.21	645.09	368.27
Right	676.21	676.21	645.09	368.27

**Table 7:** The estimated 3D coordinates of the spatial points w.r.t the base frame $O_{N}-x_{N}y_{N}z_{N}$ using ZED mini.

Point	x/mm	y/mm	z/mm	Point	x/mm	y/mm	z/mm
1	−52.97	−52.35	995.14	5	−53.83	−55.49	1493.80
2	−51.75	−104.14	1004.50	6	−47.30	−93.07	1498.50
3	−106.29	−49.44	1002.60	7	−99.29	−55.39	1478.30
4	−103.80	−112.04	989.98	8	−104.57	−99.60	1495.60
